# 2-Amino-5-ethoxy­carbonyl-4-methyl­thia­zol-3-ium chloride monohydrate

**DOI:** 10.1107/S1600536809024532

**Published:** 2009-07-04

**Authors:** Jin Rui Lin, Hong Zhao

**Affiliations:** a Ordered Matter Science Research Center, College of Chemistry and Chemical Engineering, Southeast University, Nanjing 210096, People’s Republic of China

## Abstract

In the crystal structure of the title compound, C_7_H_11_N_2_O_2_S^+^·Cl^−^·H_2_O, the cations, anions and water mol­ecules are linked by inter­molecular N—H⋯O, N—H⋯Cl, O—H⋯O and O—H⋯Cl hydrogen bonds, forming layers stacked along [20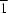].

## Related literature

For the biological activity of thia­zole derivatives, see: Turan-Zitouni *et al.* (2003 ▶). For bond-length data, see: Allen *et al.* (1987 ▶).
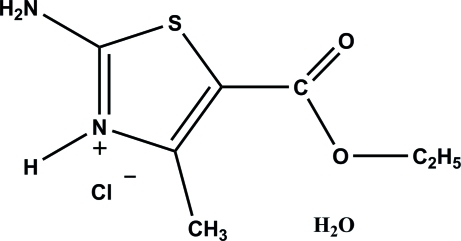


## Experimental

### 

#### Crystal data

C_7_H_11_N_2_O_2_S^+^·Cl^−^·H_2_O
*M* *_r_* = 240.70
Monoclinic, 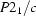

*a* = 10.637 (2) Å
*b* = 7.4463 (15) Å
*c* = 15.082 (3) Å
β = 110.22 (3)°
*V* = 1121.0 (4) Å^3^
*Z* = 4
Mo *K*α radiation
μ = 0.51 mm^−1^
*T* = 292 K
0.40 × 0.32 × 0.28 mm

#### Data collection

Rigaku SCXmini diffractometer
Absorption correction: multi-scan (*CrystalClear*; Rigaku, 2005 ▶) *T*_min_ = 0.821, *T*_max_ = 0.868
11232 measured reflections
2564 independent reflections
2097 reflections with *I* > 2σ(*I*)
*R*_int_ = 0.037

#### Refinement

*R*[*F* ^2^ > 2σ(*F* ^2^)] = 0.046
*wR*(*F* ^2^) = 0.122
*S* = 1.12
2564 reflections
133 parameters
H atoms treated by a mixture of independent and constrained refinement
Δρ_max_ = 0.48 e Å^−3^
Δρ_min_ = −0.21 e Å^−3^

### 

Data collection: *CrystalClear* (Rigaku, 2005 ▶); cell refinement: *CrystalClear*; data reduction: *CrystalClear* ; program(s) used to solve structure: *SHELXS97* (Sheldrick, 2008 ▶); program(s) used to refine structure: *SHELXL97* (Sheldrick, 2008 ▶); molecular graphics: *SHELXTL/PC* (Sheldrick, 2008 ▶); software used to prepare material for publication: *SHELXTL/PC*.

## Supplementary Material

Crystal structure: contains datablocks I, global. DOI: 10.1107/S1600536809024532/rz2337sup1.cif

Structure factors: contains datablocks I. DOI: 10.1107/S1600536809024532/rz2337Isup2.hkl

Additional supplementary materials:  crystallographic information; 3D view; checkCIF report

## Figures and Tables

**Table 1:** Hydrogen-bond geometry (Å, °)

*D*—H⋯*A*	*D*—H	H⋯*A*	*D*⋯*A*	*D*—H⋯*A*
N1—H1*A*⋯O1*W*^i^	0.86	1.94	2.789 (3)	169
N1—H1*B*⋯Cl1^ii^	0.86	2.30	3.135 (2)	164
O1*W*—H1*C*⋯Cl1	0.93	2.25	3.118 (2)	156
O1*W*—H1*D*⋯O1^iii^	0.83	2.05	2.863 (3)	167
N2—H2⋯Cl1^i^	0.79 (3)	2.35 (3)	3.141 (2)	173 (2)

Symmetry codes: (i) 
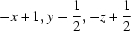
; (ii) 
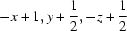
; (iii) 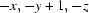.

**Fig. 1.**
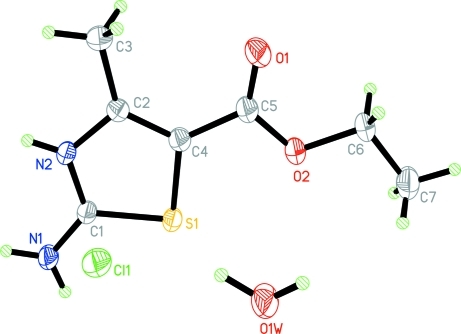
The molecular structure of the title compound, showing the atomic numbering scheme. Displacement ellipsoids are drawn at the 30% probability level.

**Fig. 2.**
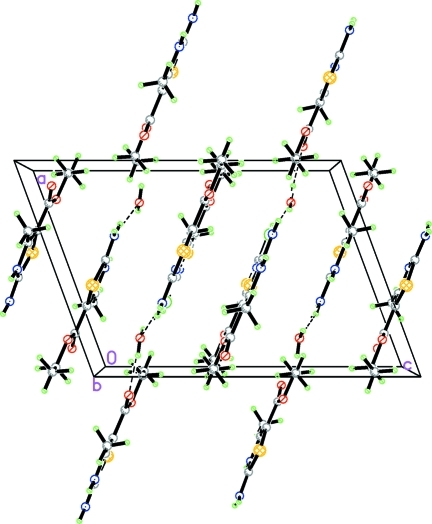
Packing diagram of the title compound, showing the structure along the b axis. Intermolecular H bonds are shown as dashed lined.

## supplementary crystallographic information

### Comment

Heterocyclic compounds containing the thiazole ring have recently received much attention for their broad-spectrum biological activities (Turan-Zitouni *et al.*, 2003). We report herein the crystal structure of the title compound.

The asymmetric unit of the title compound (Fig. 1), contains 5-(ethoxycarbonyl)-4-methylthiazol-2-aminium cations, chloride anions and water molecules in the stoichiometric ratio of 1:1:1. The cation is approximately planar, the maximum displacement being 0.062 (2) Å for atom O1. Bond lengths (Allen *et al.*, 1987) and angles have normal values. In the crystal structure (Fig. 2), cations, anions and water molecules are linked by intermolecular N—H···O, N—H···Cl, O—H···O and O—H···Cl hydrogen bonds (Table 1) to form layers stacked along [2 0 1].

### Experimental

A mixture of thiourea (0.2 mol), ethyl acetoacetate (0.1 mol) and I_2_ (0.1 mol) was stirred for 15 hours at 120°C. After refluxing the mixture with chlorhydric acid, the title compound was obtained. Colourless crystals suitable for X-ray analysis were obtained by slow evaporation of a 95% ethanol/water solution at room temperature.

### Refinement

The H2 hydrogen atom was located in a difference Fourier map and refined freely. The water H atoms were also located in a difference Fourier map but not refined [*U*_iso_(H) = 1.5*U*_eq_(O)]. All other H atoms were placed geometrically and refined as riding, with C—H = 0.96-0.97 Å, N—H = 0.86 Å, and with *U*_iso_(H) = 1.2*U*_eq_(C, N) or 1.5*U*_eq_(C) for methyl H atoms.

### Crystal data

**Table:**

C_7_H_11_N_2_O_2_S^+^·Cl^−^·H_2_O	*F*(000) = 504
*M**_r_* = 240.70	*D*_x_ = 1.426 Mg m^−^^3^
Monoclinic, *P*2_1_/*c*	Mo *K*α radiation, λ = 0.71073 Å
Hall symbol: -P 2ybc	Cell parameters from 2280 reflections
*a* = 10.637 (2) Å	θ = 2.3–27.4°
*b* = 7.4463 (15) Å	µ = 0.51 mm^−^^1^
*c* = 15.082 (3) Å	*T* = 292 K
β = 110.22 (3)°	Block, colourless
*V* = 1121.0 (4) Å^3^	0.40 × 0.32 × 0.28 mm
*Z* = 4	

### Data collection

**Table:**

Rigaku SCXmini diffractometer	2564 independent reflections
Radiation source: fine-focus sealed tube	2097 reflections with *I* > 2σ(*I*)
graphite	*R*_int_ = 0.037
Detector resolution: 13.6612 pixels mm^-1^	θ_max_ = 27.5°, θ_min_ = 3.1°
ω scans	*h* = −13→13
Absorption correction: multi-scan (*CrystalClear*; Rigaku, 2005)	*k* = −9→9
*T*_min_ = 0.821, *T*_max_ = 0.868	*l* = −19→19
11232 measured reflections	

### Refinement

**Table:**

Refinement on *F*^2^	Primary atom site location: structure-invariant direct methods
Least-squares matrix: full	Secondary atom site location: difference Fourier map
*R*[*F*^2^ > 2σ(*F*^2^)] = 0.046	Hydrogen site location: inferred from neighbouring sites
*wR*(*F*^2^) = 0.122	H atoms treated by a mixture of independent and constrained refinement
*S* = 1.12	*w* = 1/[σ^2^(*F*_o_^2^) + (0.0547*P*)^2^ + 0.5076*P*] where *P* = (*F*_o_^2^ + 2*F*_c_^2^)/3
2564 reflections	(Δ/σ)_max_ = 0.012
133 parameters	Δρ_max_ = 0.48 e Å^−^^3^
0 restraints	Δρ_min_ = −0.21 e Å^−^^3^

### Special details

**Table:**

Geometry. All e.s.d.'s (except the e.s.d. in the dihedral angle between two l.s. planes) are estimated using the full covariance matrix. The cell e.s.d.'s are taken into account individually in the estimation of e.s.d.'s in distances, angles and torsion angles; correlations between e.s.d.'s in cell parameters are only used when they are defined by crystal symmetry. An approximate (isotropic) treatment of cell e.s.d.'s is used for estimating e.s.d.'s involving l.s. planes.
Refinement. Refinement of *F*^2^ against ALL reflections. The weighted *R*-factor *wR* and goodness of fit *S* are based on *F*^2^, conventional *R*-factors *R* are based on *F*, with *F* set to zero for negative *F*^2^. The threshold expression of *F*^2^ > σ(*F*^2^) is used only for calculating *R*-factors(gt) *etc*. and is not relevant to the choice of reflections for refinement. *R*-factors based on *F*^2^ are statistically about twice as large as those based on *F*, and *R*- factors based on ALL data will be even larger.

### Fractional atomic coordinates and isotropic or equivalent isotropic displacement parameters (Å^2^)

**Table:**

	*x*	*y*	*z*	*U*_iso_*/*U*_eq_	
C1	0.5427 (2)	0.4538 (3)	0.14877 (16)	0.0386 (5)	
C2	0.3764 (2)	0.2578 (3)	0.06588 (16)	0.0388 (5)	
C3	0.3260 (3)	0.0717 (3)	0.0413 (2)	0.0542 (7)	
H3A	0.2446	0.0563	0.0545	0.081*	
H3B	0.3920	−0.0121	0.0781	0.081*	
H3C	0.3088	0.0508	−0.0247	0.081*	
C4	0.3170 (2)	0.4157 (3)	0.03211 (17)	0.0411 (5)	
C5	0.1833 (2)	0.4419 (3)	−0.03804 (17)	0.0455 (6)	
C6	0.0280 (3)	0.6581 (4)	−0.1270 (2)	0.0616 (8)	
H6A	−0.0419	0.6052	−0.1079	0.074*	
H6B	0.0199	0.6116	−0.1888	0.074*	
C7	0.0156 (3)	0.8556 (5)	−0.1302 (3)	0.0850 (11)	
H7A	0.0248	0.9001	−0.0686	0.128*	
H7B	−0.0706	0.8889	−0.1742	0.128*	
H7C	0.0846	0.9063	−0.1501	0.128*	
Cl1	0.33656 (7)	0.42792 (8)	0.29075 (5)	0.0540 (2)	
N1	0.65955 (19)	0.5069 (3)	0.20741 (15)	0.0499 (5)	
H1A	0.7182	0.4290	0.2380	0.060*	
H1B	0.6775	0.6197	0.2153	0.060*	
N2	0.5037 (2)	0.2825 (2)	0.13088 (14)	0.0391 (4)	
O1	0.10510 (19)	0.3235 (3)	−0.07219 (16)	0.0720 (6)	
O2	0.15910 (17)	0.6150 (2)	−0.05873 (13)	0.0544 (5)	
O1W	0.1705 (2)	0.7502 (3)	0.17716 (18)	0.0799 (7)	
H1C	0.2076	0.6373	0.1937	0.120*	
H1D	0.0879	0.7443	0.1503	0.120*	
S1	0.41995 (6)	0.59887 (7)	0.08238 (4)	0.04234 (19)	
H2	0.547 (2)	0.198 (3)	0.1547 (16)	0.033 (6)*	

### Atomic displacement parameters (Å^2^)

**Table:**

	*U*^11^	*U*^22^	*U*^33^	*U*^12^	*U*^13^	*U*^23^
C1	0.0338 (11)	0.0311 (10)	0.0441 (12)	0.0043 (8)	0.0047 (9)	0.0015 (9)
C2	0.0357 (11)	0.0341 (11)	0.0414 (11)	−0.0007 (9)	0.0067 (9)	0.0003 (9)
C3	0.0496 (15)	0.0364 (12)	0.0625 (16)	−0.0065 (11)	0.0014 (12)	0.0003 (11)
C4	0.0343 (11)	0.0361 (11)	0.0463 (12)	0.0002 (9)	0.0056 (9)	0.0017 (10)
C5	0.0342 (12)	0.0447 (13)	0.0498 (13)	0.0033 (10)	0.0048 (10)	0.0023 (11)
C6	0.0406 (14)	0.0564 (16)	0.0654 (17)	0.0121 (12)	−0.0103 (12)	−0.0003 (13)
C7	0.0572 (19)	0.0549 (18)	0.110 (3)	0.0138 (15)	−0.0128 (18)	0.0051 (18)
Cl1	0.0531 (4)	0.0343 (3)	0.0627 (4)	−0.0034 (2)	0.0047 (3)	−0.0010 (3)
N1	0.0366 (10)	0.0341 (10)	0.0610 (13)	0.0021 (8)	−0.0061 (9)	0.0015 (9)
N2	0.0359 (10)	0.0284 (9)	0.0443 (10)	0.0058 (8)	0.0028 (8)	0.0030 (8)
O1	0.0430 (11)	0.0534 (12)	0.0924 (15)	−0.0044 (9)	−0.0112 (10)	0.0004 (11)
O2	0.0409 (9)	0.0456 (10)	0.0582 (10)	0.0083 (7)	−0.0064 (8)	0.0013 (8)
O1W	0.0451 (11)	0.0465 (11)	0.1127 (17)	−0.0019 (9)	−0.0180 (11)	−0.0051 (11)
S1	0.0357 (3)	0.0299 (3)	0.0515 (3)	0.0048 (2)	0.0024 (2)	0.0036 (2)

### Geometric parameters (Å, °)

**Table:**

C1—N1	1.313 (3)	C6—O2	1.454 (3)
C1—N2	1.339 (3)	C6—C7	1.476 (4)
C1—S1	1.724 (2)	C6—H6A	0.9700
C2—C4	1.348 (3)	C6—H6B	0.9700
C2—N2	1.382 (3)	C7—H7A	0.9600
C2—C3	1.486 (3)	C7—H7B	0.9600
C3—H3A	0.9600	C7—H7C	0.9600
C3—H3B	0.9600	N1—H1A	0.8600
C3—H3C	0.9600	N1—H1B	0.8600
C4—C5	1.463 (3)	N2—H2	0.79 (3)
C4—S1	1.750 (2)	O1W—H1C	0.9259
C5—O1	1.199 (3)	O1W—H1D	0.8324
C5—O2	1.330 (3)		
			
N1—C1—N2	125.3 (2)	C7—C6—H6A	110.3
N1—C1—S1	123.57 (17)	O2—C6—H6B	110.3
N2—C1—S1	111.12 (16)	C7—C6—H6B	110.3
C4—C2—N2	111.61 (19)	H6A—C6—H6B	108.5
C4—C2—C3	129.6 (2)	C6—C7—H7A	109.5
N2—C2—C3	118.8 (2)	C6—C7—H7B	109.5
C2—C3—H3A	109.5	H7A—C7—H7B	109.5
C2—C3—H3B	109.5	C6—C7—H7C	109.5
H3A—C3—H3B	109.5	H7A—C7—H7C	109.5
C2—C3—H3C	109.5	H7B—C7—H7C	109.5
H3A—C3—H3C	109.5	C1—N1—H1A	120.0
H3B—C3—H3C	109.5	C1—N1—H1B	120.0
C2—C4—C5	126.9 (2)	H1A—N1—H1B	120.0
C2—C4—S1	112.00 (17)	C1—N2—C2	115.39 (18)
C5—C4—S1	121.05 (17)	C1—N2—H2	125.1 (18)
O1—C5—O2	124.2 (2)	C2—N2—H2	119.5 (18)
O1—C5—C4	124.7 (2)	C5—O2—C6	116.1 (2)
O2—C5—C4	111.1 (2)	H1C—O1W—H1D	111.3
O2—C6—C7	107.3 (2)	C1—S1—C4	89.88 (11)
O2—C6—H6A	110.3		

### Hydrogen-bond geometry (Å, °)

**Table:**

*D*—H···*A*	*D*—H	H···*A*	*D*···*A*	*D*—H···*A*
N1—H1A···O1W^i^	0.86	1.94	2.789 (3)	169
N1—H1B···Cl1^ii^	0.86	2.30	3.135 (2)	164
O1W—H1C···Cl1	0.93	2.25	3.118 (2)	156
O1W—H1D···O1^iii^	0.83	2.05	2.863 (3)	167
N2—H2···Cl1^i^	0.79 (3)	2.35 (3)	3.141 (2)	173 (2)

Symmetry codes: (i) −*x*+1, *y*−1/2, −*z*+1/2; (ii) −*x*+1, *y*+1/2, −*z*+1/2; (iii) −*x*, −*y*+1, −*z*.
